# Evidence of Cerebellar Involvement in the Onset of a Manic State

**DOI:** 10.3389/fneur.2018.00774

**Published:** 2018-09-12

**Authors:** Michela Lupo, Giusy Olivito, Libera Siciliano, Marcella Masciullo, Marco Molinari, Mara Cercignani, Marco Bozzali, Maria Leggio

**Affiliations:** ^1^Ataxia Laboratory, IRCCS Fondazione Santa Lucia, Rome, Italy; ^2^Neuroimaging Laboratory, IRCCS Fondazione Santa Lucia, Rome, Italy; ^3^Ph.D. Program in Behavioral Neuroscience, Sapienza University of Rome, Rome, Italy; ^4^SPInalREhabilitation Lab, IRCCS Fondazione Santa Lucia, Rome, Italy; ^5^Robotic Neurorehabilitation Lab, Neurorehabilitation 1 and Spinal Center, IRCCS Fondazione Santa Lucia, Rome, Italy; ^6^Clinical Imaging Science Center, Brighton and Sussex Medical School, Brighton, United Kingdom; ^7^Department of Psychology, Sapienza University of Rome, Rome, Italy

**Keywords:** mania, cerebellum, MRI, bipolar disorder, mood

## Abstract

We described the cerebello-cerebral functional connectivity in a subject who developed a manic state after a cerebellar lesion. Whole brain investigation, performed by means of an advanced MRI examination, evidenced an isolated lesion involving the left lobules VI, VIIa (crus I), and IX and the posterior area of the vermis. The cerebello-cerebral functional connectivity analysis detected a pattern of altered connectivity in specific areas of the prefrontal-striatal-thalamic circuits that are typically altered in bipolar subjects during the manic state. Specifically, a pattern of hypo-connectivity was found between the cerebellum and cerebral regions known to be implicated in emotion modulation and social interaction. Conversely, a pattern of hyper-connectivity was found between the cerebellum and posterior cerebral cortical regions that are involved in sensorimotor functions. The present study represents the first evidence that dysregulation of cerebral networks consequent to a cerebellar lesion is at the root of bipolar disorder, at least the manic state, and provides a new framework for interpreting cerebellar modulation in the regulation of mood in specific psychiatric conditions.

## Introduction

Mania is a period of 1 week or more in which a person's normal behaviour changes and includes a euphoric state and is a condition typical of bipolar disorder. Although there is increasing evidence that the cerebellum is connected to cortical areas involved in the pathophysiology of psychiatric disorders (1–3), no studies have specifically investigated the cause-effect relationship between cerebellar damage and the development of emotional dysregulation in terms of manic/depressive mood.

However, in the last 20 years, increasing evidence has changed the view of the cerebellum from a structure specifically implicated in motor control to a structure involved in higher-order cognitive and emotional functions (4–6).

Moreover, the clinical description of the Cerebellar Cognitive Affective Syndrome (CCAS) (6) allowed to define a constellation of behavioral and cognitive symptoms consequent of a cerebellar pathology (6).

Furthermore, neuroimaging studies have demonstrated the existence of anatomical correlations, organized in reciprocal loops, between the sensorimotor and association areas of the cerebral cortex and distinct anatomical and functional cerebellar regions (7–9).

These studies have demonstrated that the anterior cerebellar lobes (lobules I/II through V) are connected with the sensorimotor cortices, while the posterior cerebellar lobes (lobules VI through IX) are connected with the association cortices (10–13). Furthermore, the posterior vermis, including parts of lobule IX, is connected with limbic networks implicated in emotional and behavioural processing (14). This part of the cerebellum has been defined as the “limbic cerebellum” (15).

Despite these connections between the limbic cerebellum and the well-known limbic cerebral networks, only very few studies on patients affected by mood swings have addressed the cerebellar role in mood alterations (16).

Taking into account all these considerations, it is possible to hypothesize that a lesion in the limbic cerebellum plays a key role in affecting mood and behaviour.

The aim of the present study was to provide some insights into this issue, showing that the disruption of specific cerebello-cortical circuits can cause the development of mood disorders.

To test this hypothesis, we analysed cerebello-cerebral connectivity in a patient who suffered a severe manic mood state after a cerebellar accident.

Taking into account the aim of the study, the dentate nucleus (DN) was chosen as region of interest (ROI) for the seed-based analysis. Indeed, the cerebellar information ultimately converges on Purkinje neurons and is, then, funneled out through the neurons of the DN, through which the cerebellum communicates with the other parts of central nervous system. Thus, when the Purkinje cells are affected as consequence of a cerebellar cortical lesion, the connections between the DN, which represents the sole output of the cerebellar cortex, and its target regions may be also altered.

### Case report

MT is a 43-year-old right-handed woman who used to work as a lawyer. At the age of 42 years, she had suffered a rupture of the cerebellar arteriovenous malformation (AVM), which was treated with embolization (July 2014). Ten months after the lesion (May 2015), she was admitted to the Ataxia Laboratory of IRCCS Fondazione Santa Lucia. A neurological examination revealed severe ataxia with a total motor score of 46/100 on the International Cooperative Ataxia Rating Scale (ICARS) (17).

During the anamnestic interview, no cognitive problems were apparent prior to the cerebellar lesion. However, episodes of inappropriate behaviours were described in childhood, although they had been underestimated by MT's parents. The patient's major complaint was the worsening of some symptoms (i.e., impulsiveness) and the onset of other behavioural abnormalities, also confirmed by the husband, and included referred hallucinations, together with a euphoric state similar to a manic mood phase. These symptoms arose after the cerebellar accident; they were already present in September 2014 (5 months after the acute event) and worsened over time.

Therefore, an assessment of her personality and mood changes was performed by an expert psychotherapist using the Structured Clinical Interview for DSM IV Axis I Disorders (SCID I) (18) and the Structured Clinical Interview for DSM IV Axis II Disorders (SCID-II) (19). It should be noted that, at the time of the patient's evaluation, the Italian version of SCID I and SCID II scale related to the new DSM- 5 criteria (20) was not yet available. It was determined that MT suffered from borderline personality organization and bipolar I disorder, mixed episode, as already described by Lupo et al. (21).

Moreover, an adjustment disorder with a disturbance in conduct, from which she had been suffering since childhood, was also diagnosed. In Lupo et al. (21), the authors linked these behavioural alterations to an abnormal cerebellar influence during cerebral development due to the congenital nature of the AVM (21).

The patient was never pharmacologically treated for psychiatric symptomatology.

The patient's major psychiatric symptoms, as detected by the psychological assessment, and experienced during the manic mood phase, are listed below:

- Euphoria
- Disinhibited and inadequate behaviour (e.g., laughter during funerals)
- Impairment in social interaction
- Impulsiveness (e.g., spending money irresponsibly)
- Aggressiveness
- Transient stress-related psychotic-like symptoms (e.g., hallucinations and dissociative symptoms)
- Emotional lability and mood swings (e.g., from a mild depression to a euphoric mood state)
- Alexithymia

**Figure 1:**
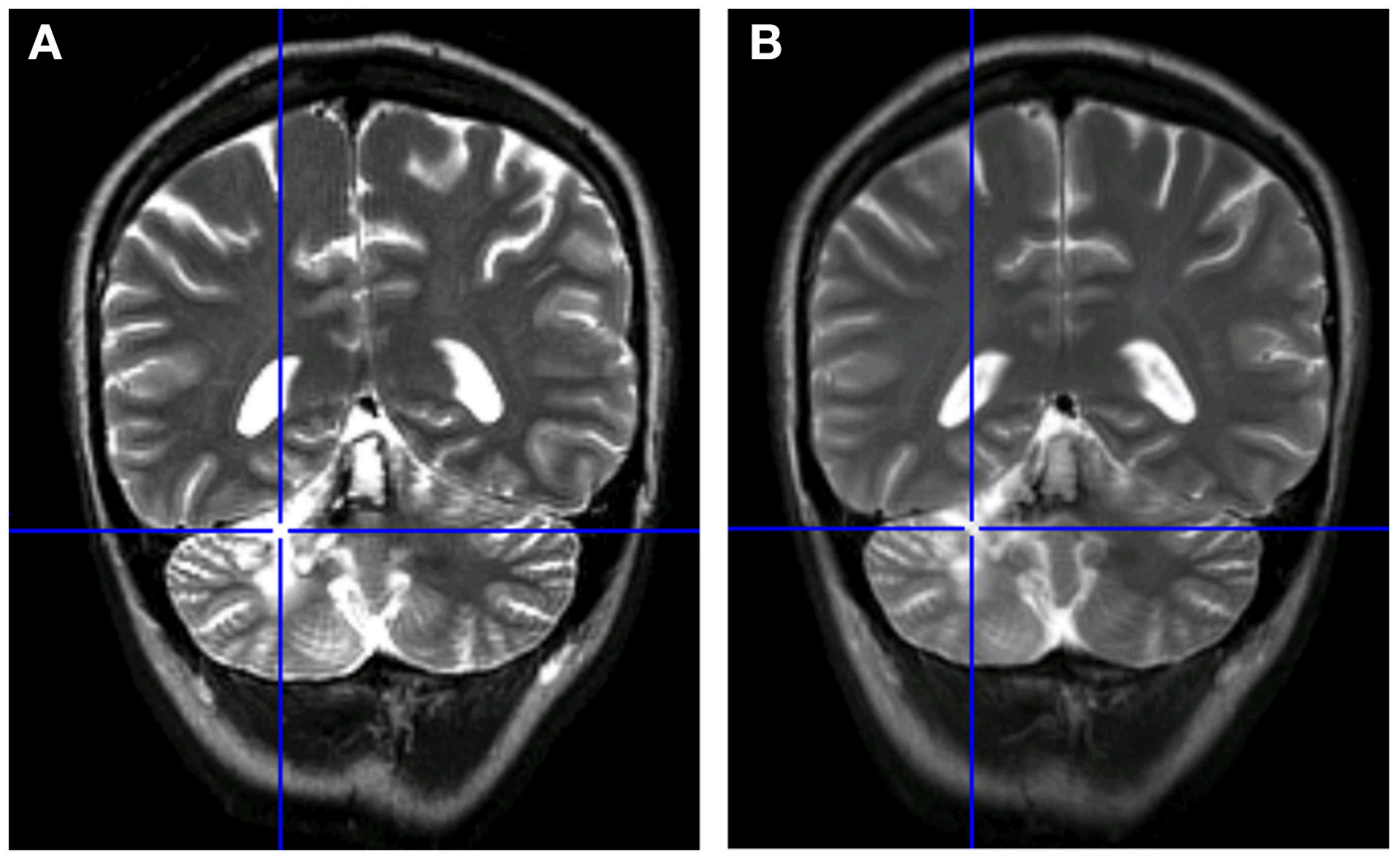
Comparison between the patient's MRI scans. **(A)** MRI in May 2015 (10 months after acute event of July 2014). **(B)** MRI at follow-up in January 2016 (8 months after the initial scan).

At the time of psychological assessment (May 2015), a whole brain investigation was performed by means of an advanced MRI examination. MT's lesion affected the left hemispheric regions of the cerebellum, specifically lobules VI, VIIa (crus I), and IX, and the posterior area of the vermis, with a sparing of dentate nucleus (total lesion extent: 3934 mm3). No other cortical or subcortical lesions were detected (see Figure 1A).

The selective cerebellar lesion was also confirmed by examination of tomography images (PET).

Furthermore, in January 2016, an MRI follow-up did not show any modification in comparison with the scan from 8 months before (see Figure 1B).

## Methods

### MRI data acquisition protocol

For the MRI analysis, 20 age-matched females with no history of psychiatric or neurological illness were enrolled as the control group [mean age (52.45), standard deviation (5.75)] (see Table 1 for details). A significance test (24) ensured that there was no difference between MT and control sample age (*t* = −1,43; *p* = 0.087).

**Table 1:** Demographic characteristics of MT and the control group.

**Group**	**N^°^**	**Age**	**Education (years)**	**Gender**	**IQ (cut off: < 70)**	**ICARS motor score**
MT	1	43	18	F	105	46/100
Cnt	20	52.4 (5.75)	–	F	–	–

*Cnt, Control Group; F, female; IQ, Intelligence Quotient assessed by Wechsler Adult Intelligent Scale-Revised (WAIS-R) (22, 23). Age data are reported as the mean and standard deviation (SD)*.

Both the patient and the controls underwent an MRI examination at 3T (Magnetom Allegra, Siemens, Erlangen, Germany) that included the following acquisitions: (1) dual-echo turbo spin echo [TSE] (TR = 6190 ms, TE = 12/109 ms); (2) fast-FLAIR (TR = 8170 ms, 204TE = 96 ms, TI = 2100 ms) for conventional MRI visualization of the brain; (3) 3D modified driven equilibrium fourier transform (MDEFT) scan (TR = 1338 ms, TE = 2.4 ms, matrix = 256 × 224 × 176, in-plane FOV = 250 × 250 mm^2^, slice thickness = 1 mm) for structural T1-weighted imaging of the brain; and (4) T2^*^ weighted echo planar imaging (EPI) sensitized to the blood oxygenation dependent imaging (BOLD) contrast (TR: 2080 ms, TE: 30 ms, 32 axial slices parallel to AC-PC line, matrix: 64 × 64, pixel size: 3 × 3 mm^2^, slice thickness: 2.5 mm, flip angle: 70°) for resting state fMRI. BOLD echo planar images were collected during rest for a 7 min and 20 s period, resulting in a total of 220 volumes. To characterize the brain anatomy and to ensure the absence of macroscopic extracerebellar abnormality, the TSE and FLAIR scans of patient MT, acquired as part of the MRI protocol, were inspected by an expert neuroradiologist.

According to the inclusion criteria, conventional MRI scans of control subjects were also reviewed to exclude any pathological conditions affecting the brain.

### Resting state fMRI data preprocessing

Data were pre-processed using Statistical Parametric Mapping [Wellcome Department of Imaging Neuroscience; SPM8 (http://www.fil.ion.ucl.ac.uk/spm/)] and in-house software implemented in MATLAB (The Mathworks Inc., Natick, Massachusetts, USA). For each subject, the first four volumes of the fMRI series were discarded to allow for T1 equilibration effects. The pre-processing steps included correcting for head motion, compensating for slice-dependent time shifts, normalizating to the EPI template in Montreal Neurological Institute (MNI) coordinates provided with SPM8, and smoothing with a 3D Gaussian Kernel with 8 mm^3^ full-width at half maximum. For each data set, motion correction was checked to ensure that the maximum absolute shift did not exceed 2 mm and the maximum absolute rotation did not exceed 1.5°. The global temporal drift was removed using a 3rd-order polynomial fit, and the signal was regressed against the realignment parameters and the signal averaged over whole brain voxels to remove other potential sources of bias. Then, all images were filtered by a phase-insensitive bandpass filter (pass band 0.01–0.08 Hz) to reduce the effect of low-frequency drift and high-frequency physiological noise.

### Seed-based analyses

**Figure 2:**
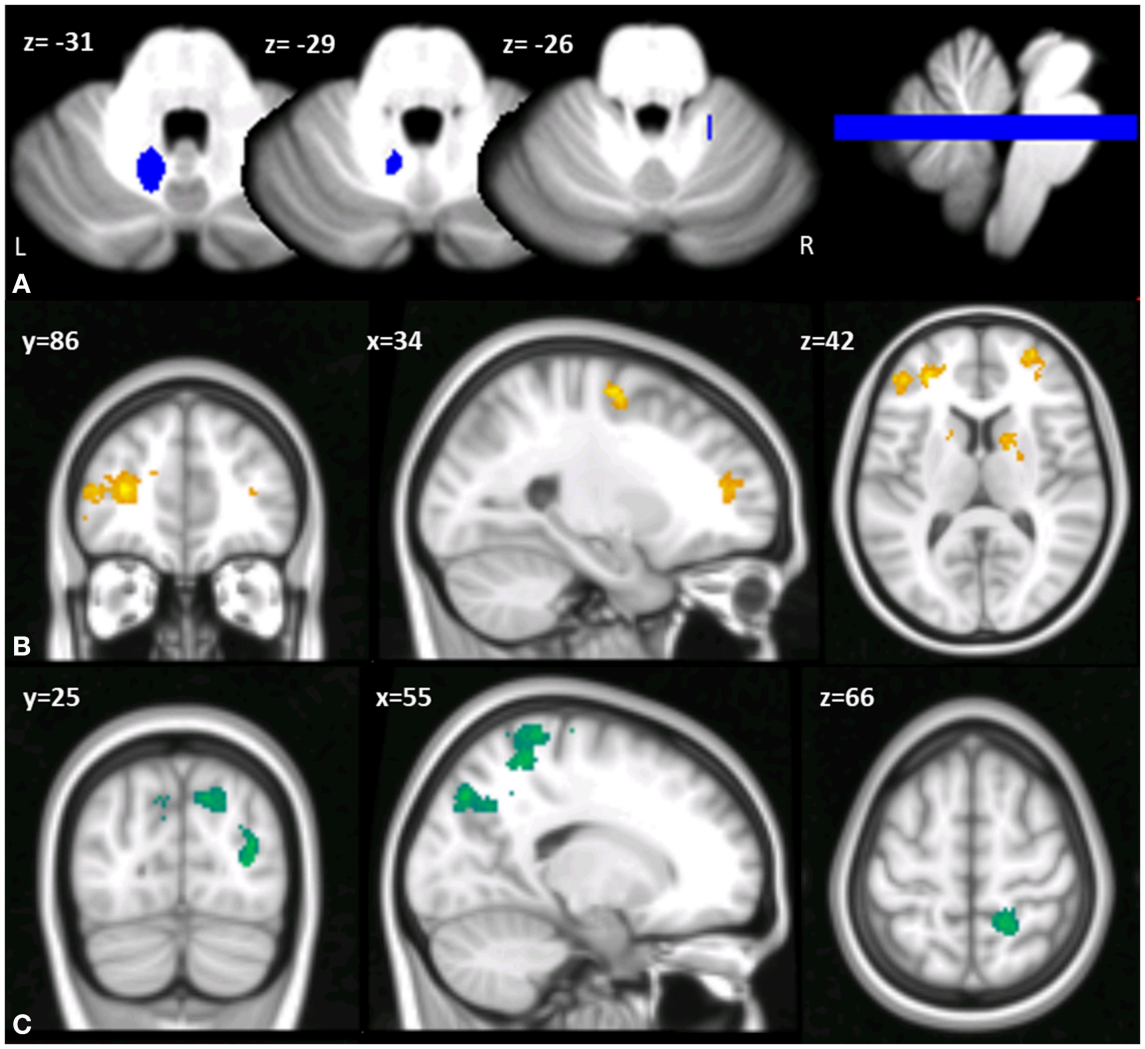
**(A)** Multislice axial (z) view of the left DN mask superimposed on the spatially unbiased atlas template of the cerebellum and brainstem (SUIT) (25). L, left; R, right. **(B,C)** Cerebello-cerebral functional connectivity differences in MT compared to the control group. Clusters of significant hypo-connectivity **(B)** and hyper-connectivity **(C)** with cerebellar DN are shown in coronal (y), sagittal (x), and axial slices (z). X, Y, Z in the MNI space. Results considered significant at *p* < 0.001 uncorrected after FWE correction at the cluster level.

Based on the side of the lesion, the left DN was chosen as the ROI for the seed-based analysis. Thus, a left DN mask was extracted with reference to the spatially unbiased atlas template of the cerebellum and brainstem (SUIT) (25) (Figure 2A) and resliced into EPI standard space.

The mean time course of the voxels within the chosen ROI was extracted for every participant and used as a regressor in a 1st-level SPM analysis, thus extracting the voxels in the whole brain showing a significant correlation with the DN. At the second level, a two-sample *t*-test model was used to explore differences in connectivity between the left DN and the rest of the brain in the patient compared to the controls, assuming equality of variance between the groups.

Between-group statistical significance was set at *p* < 0.05 FWE-corrected at the cluster level (clusters formed with uncorrected voxels *p* < 0.001 at the cluster level).

## Results

When comparing the pattern of left DN functional connectivity in the patient against the control group, a pattern of altered FC of the left DN with regions of both left and right cerebral cortex was apparent (see Tables 2, 3 for details). Specifically, a large cluster of hypo-connectivity was found between the left DN and the anterior cerebral cortical regions with peak voxels of significant hypo-connectivity centered in the bilateral frontal pole, including the dorsolateral prefrontal cortex and the orbitofrontal cortex, the left superior frontal gyrus, the left paracingulate and anterior cingulate cortex, and the caudate nucleus (see Figure 2B). Conversely, a pattern of hyper-connectivity was found between the left DN and posterior cerebral cortical regions, with peak voxels of significant hyper-connectivity centered in the right superior parietal lobule, right post-central gyrus and bilateral lateral occipital cortex (Figure 2C).

**Table 2:** Functional hypo-connectivity results between the DN and cerebral cortex regions.

**Cluster size (NoV)**	**Coordinates**			**Cluster peak *Z*-score**	**Brain region**
	**x**	**y**	**z**		
625	−14	34	22	4.96	L-Paracingulate cortex
	−8	16	28	4.62	L-Cingulate cortex
	−30	40	12	4.58	L-Frontal Pole
150	30	56	8	5.12	R-Frontal Pole
	36	50	10	3.42	
	32	42	10	3.33	
232	16	10	16	4.45	Caudate
147	−24	−8	60	4.33	L-Superior Frontal Gyrus
	−18	−14	42	3.99	
	−18	−2	52	3.88	

*MNI coordinate space (x, y, z) and peak Z-score of the peak voxels showing the greatest significant differences in a cluster are reported. Only regions that survived after correction for multiple comparisons (FWE corrected p < 0.05, clusters formed with uncorrected voxels p < 0.001 at cluster level) were considered. NoV, number of voxels; L, left; R, right*.

**Table 3:** Functional hyper-connectivity results between the DN and cerebral cortex regions.

**Cluster size (NoV)**	**Coordinates**			**Cluster peak *Z*-score**	**Brain region**
	**x**	**y**	**z**		
176	34	−76	12	4.54	L-Lateral Occipital Cortex
220	−6	−82	30	4.52	L-Cuneal cortex
	−14	−86	18	4.32	L-Lateral Occipital cortex
	−19	−92	30	4.00	L-Occipital pole
377	16	−40	72	4.50	R-Post-central gyrus
	20	−46	56	4.22	R-Superior Parietal lobe
	14	−24	72	4.00	R-Precentral gyrus
251	20	−76	42	4.33	R-Lateral Occipital cortex
	22	−62	34	3.96	R-Precuneus
	10	−82	38	3.79	R-Cuneal cortex

*MNI coordinate space (x, y, z) and peak Z-score of the peak voxels showing the greatest significant differences in a cluster are reported. Only regions that survived after correction for multiple comparisons (FWE corrected p < 0.05, clusters formed with uncorrected voxels p < 0.001 at cluster level) were considered. NoV, number of voxels; L, left; R, right*.

A detailed report of the seed-based analyses with MNI coordinates and peak-z scores is summarized in Tables 2, 3.

## Discussion

Through an in-depth neuroimaging data analysis, the present study aimed to demonstrate the mechanism through which a cerebellar lesion may affect mood states.

According to the CCAS (6), it is known that the dysregulation of affect and behaviour occurs mainly when lesions involve the limbic cerebellum (15). Indeed, in a meta-analysis by Stoodley and Schmahmann (12), the authors showed that the posterior lobes VI and VII, including crus I and crus II, are specifically involved in cognitive and emotional functions (12). In particular, damage to the posterior vermis, including parts of lobule IX, is most often associated with emotional lability, flattened affect, and disinhibited behaviour (12).

Thus, considering MT's lesion, we found strong associations between our findings and the anatomo-functional topography previously described. Indeed, MT's lesion affected the left lobules VI, VIIa (crus I), and IX and the posterior area of the vermis.

Analysing the patient's FC data, the rs-fMRI showed an hypo-connectivity between the left DN and regions in the frontal pole (i.e., the dorsolateral prefrontal cortex, orbitofrontal cortex, superior and middle frontal gyrus), paracingulate cortex (the anterior cingulate cortex), and caudate nucleus (Figure 2B).

Conversely, a pattern of hyper-connectivity was found between the left DN and posterior cerebral cortical regions (Figure 2C), which are involved in sensorimotor functions.

Considering the cluster of regions in which we found hypo-connectivity in MT, it has to be highlighted that these regions are known to be implicated in cognition, emotion modulation, regulation of affective states and social interaction both in bipolar and borderline patients (26, 27). Furthermore, similar hypo-connectivity changes have been evidenced in several regions of the prefrontal cortex and striatum during the manic state in bipolar subjects (27–29). In particular, two distinct cortico-subcortical networks have been linked to the depressive and manic state conditions of bipolar disorder (16). Specifically, manic states are commonly associated with decreased activation in the ventral prefrontal cortex, anterior cingulate, and striatum, consistent with the hypothesis of a loss of ventral prefrontal modulation of the limbic brain during mania (30, 31).

Within this framework, the study of our patient's functional connectivity may advance interesting insights. Indeed, MT's manic state is probably caused by the structural changes following the cerebellar lesion that have modified the functional connectivity between the cerebellum and the cortico-subcortical networks specifically involved in the manic state (16, 26, 31, 32).

Moreover, our results are in line with the fMRI data of Shaffer et al. (16) that found a functional activity reduction in the cerebellar vermis and in the left cerebellar hemisphere more pronounced in the manic group, suggesting the cerebellum as a possible key region for the regulation of the manic episodes (16). The similarity between functional activations in the manic group of Shaffer et al. (16) and MT's cerebello-cortical FC alterations is congruent with the manic mood of our patient.

In a previous work (21), we described the case of MT, proposing a link between cerebellar lesion, and the presence of behavioural disturbances from childhood that merged into a personality disorder after the cerebellar accident in adulthood. In the present study, we demonstrate a dysregulation of the cerebello-cerebral network in specific areas that are known to be altered in the manic state in bipolar patients (16, 26, 30–32). Because the mood disorder appeared after the cerebellar lesion in our patient, and her rs-fMRI evidenced an impaired functional connectivity between the cerebellum and the same areas affected during the manic phase in bipolar patients (16), this evidence proves for the first time the association between cerebellar FC alterations and the onset of a manic state.

## Conclusions

The present study demonstrates an association between aberrant cerebello-cortical FC and the onset of a manic state. Specifically, our results show that there is an overlap between the areas of the prefrontal-striatal-thalamic circuits that are altered in the manic state of bipolar disorder and the regions with impaired functional connectivity in our patient that presented a manic state after an isolated cerebellar lesion.

Taken together, these results help to fill the gap in the mechanisms through which cerebellar modulation may regulate mood state in specific psychiatric conditions.

## Ethics statement

The experimental procedures were approved by the Ethics Committee of IRCSS Santa Lucia Foundation. Written informed consent was obtained from each subject per the Helsinki Declaration. Written informed consent was obtained by the patient for the publication of this case report.

## Author contributions

MLu drafting and revising the manuscript, study concept and design, analysis and interpretation of data. Accepts responsibility for conduct research and final approval, acquisition of data, study supervision. GO drafting and revising the manuscript, acquisition, and analysis of MRI data. LS drafting and revising the manuscript, acquisition of data. MMa drafting and revising the manuscript. MMo drafting and revising the manuscript. MC supervision of MRI analysis and revising the manuscript. MB supervision of MRI analysis and revising the manuscript. MLe drafting and revising the manuscript, study concept and design, analysis and interpretation of data, accepts responsibility for conduct research and final approval, study supervision.

### Conflict of interest statement

The authors declare that the research was conducted in the absence of any commercial or financial relationships that could be construed as a potential conflict of interest.
